# Vasitis: a rare diagnosis mimicking inguinal hernia: a case report

**DOI:** 10.1186/s12894-019-0460-x

**Published:** 2019-04-29

**Authors:** Chih-Wei Chen, Chin-Ho Lee, Tsung-Yi Huang, Yi-Ming Wang

**Affiliations:** 1 Department of Radiology, Kaohsiung Medical University Hospital, Kaohsiung Medical University, No. 100, Ziyou 1st Rd., Sanmin District, Kaohsiung City, 80756 Taiwan, Republic of China; 2 Department of Urology, Kaohsiung Medical University Hospital, Kaohsiung Medical University, Kaohsiung City, Taiwan

**Keywords:** Hernia, Vasitis, Vas deferens, Inguinal hernia

## Abstract

**Background:**

Vasitis or inflammation of the vas deferens is a rare condition, and few case reports with computed tomography images have been published since 1980.

**Case presentation:**

A 50-year-old man presented with severe right inguinal and lower abdominal pain. Initial diagnosis at the emergency department was incarcerated or strangulated inguinal hernia. The computed tomography scan revealed diffuse edematous changes of right spermatic cord and vas deferens with peripheral fat stranding. Correlating with his clinical symptoms, signs, and imaging findings, the diagnosis of vasitis was made. We report a case of acute vasitis about the cause, symptom, pathogen, differential diagnoses, image findings, and treatment.

**Conclusion:**

Although very rare, vasitis should be listed as one of the differential diagnosis for inguinal mass lesions. Cross-sectional imaging may be necessary to confirm the diagnosis and exclude differentials such as an inguinal hernia. Recognition of the characteristic image findings can help to make the correct diagnosis and avoid unnecessary surgery.

## Background

Acute vasitis or inflammation of the vas deferens is an extremely rare disease, with only about twenty adult case reports published since 1980. Few of these contain computed tomography (CT) images [1–10]. Clinically, it presents with nonspecific symptoms of inguinal swelling and local pain that can mimic orchitis, epididymitis, testicular torsion, and inguinal hernia. Familiarity with symptoms, image findings, and differential diagnoses is essential to prevent unnecessary surgery, especially when symptoms mimic an inguinal hernia.

## Case presentation

**Fig. 1:**
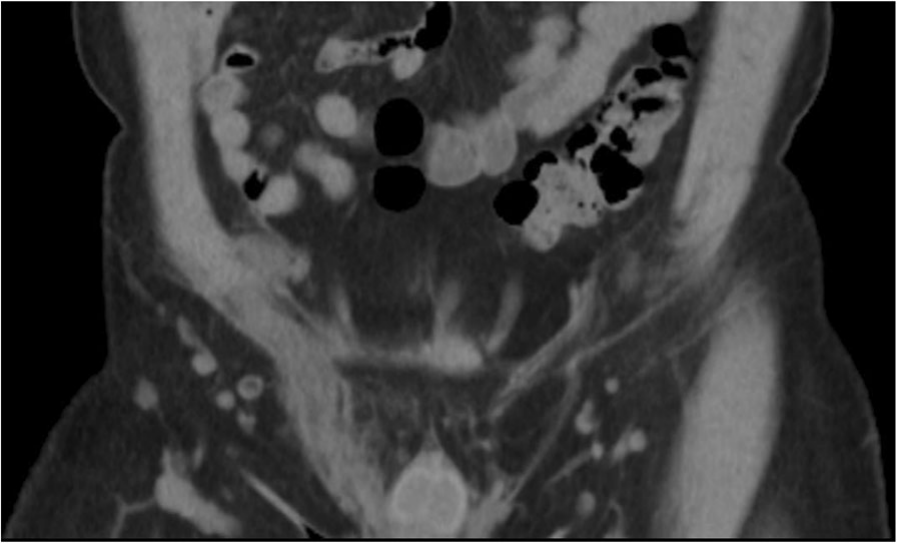
Non-enhanced coronal CT image revealed diffuse edematous change of right spermatic cord (white arrow)

**Fig. 2:**
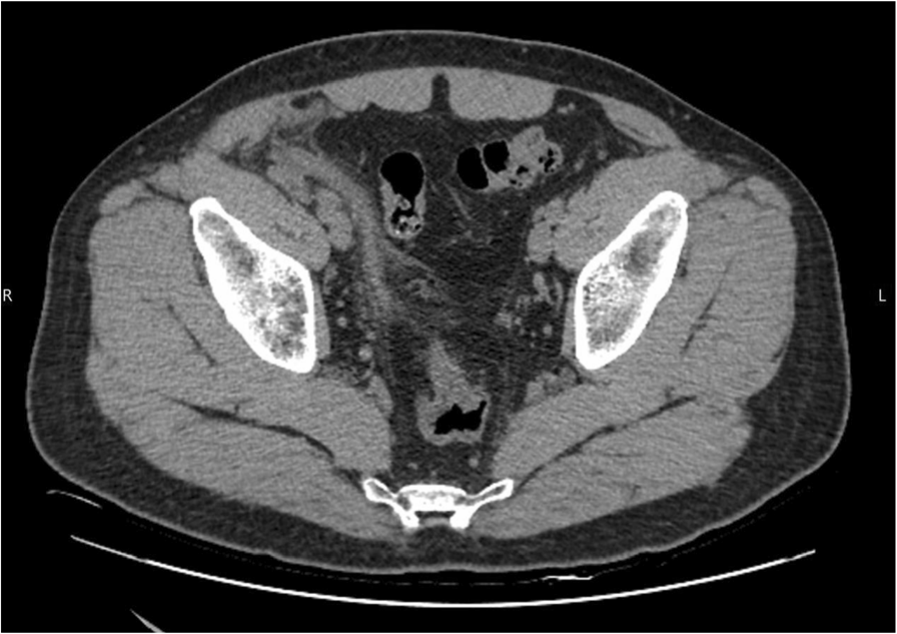
Non-enhanced axial CT image revealed dilated right vas deferens with peripheral fat stranding (white arrows)

A middle-aged male presented to our emergency department with severe right inguinal and lower abdominal pain, exaggerated by walking and Valsalva’s maneuver. He did not have a fever or chillness. The patient has no other prior medical history, no trauma history, no recent travel history, and no habitual alcohol and cigarette consumption. The patient is of Han Chinese origin and is currently working as an office clerk. He denied any family history of similar symptoms, no recent heavy lifting activities, and no high-risk sexual behaviors. The physical examination revealed tenderness and swelling in the right inguinal area. The laboratory tests revealed leukocytosis (11.39 × 103 /uL), elevated C reactive protein (35.4 mg/L) and leukocyturia, but a negative result of the urine culture. Due to the progression of localized right inguinal pain, the emergency doctor arranged computed tomography scan to rule out incarcerated inguinal hernia. The scan revealed diffuse edematous changes of right spermatic cord and vas deferens with peripheral fat stranding and no herniated bowel loop [Figs. 1, 2]. Correlating the patient’s clinical symptoms, signs, and, imaging findings, the emergency doctor was able to overturn the initial diagnosis of an inguinal hernia and confirm vasitis. Empirical antibiotic treatment (Levofloxacin) was prescribed with good response.

### Timeline

**Table:** 

Dates	Patient Relevant Past Medical History and Interventions		
No previously medical or surgical history. No family medical history.			
Dates	Summaries from Initial Emergency and Follow-up Visits	Diagnostic Testing	Interventions
2017-Feb	Came to the emergency department due to severe right inguinal and lower abdominal pain.	Physical examination: Right inguinal mass with pain, exaggerated when walking and Valsalva’s maneuver.	Prescribed oral antibiotic use: 500 mg of Levofloxacin (Cravit) daily for two weeks.
		laboratory data: leukocytosis, elevated C reactive protein and leukocyturia,	
		CT: Revealed unilateral edematous spermatic cord without evidence of herniated bowel loop.	
7 Days later	Out-patient department follow-up.	Symptoms improved.	
14 Days later	Out-patient department follow-up.	Symptoms improved.	

## Discussion and Conclusions

Inflammation of the vas deferens or vasitis is a rare condition categorized by Chan and Schlegel as acutely painful infective vasitis or asymptomatic vasitis nodosa [8]. Vasitis nodosa was first described in 1943 by Benjamin [9] as asymptomatic, chronic inflammatory reactions associated with blockage of the vas deferens, which causes high intra-luminal pressure with spermatozoa leakage and inflammatory process. Clinical presentation is with asymptomatic nodular lesions in the vas deferens, and most cases have a history of vasectomy. No specific treatment is required [4].

Acute infective vasitis is thought to be due to the retrograded spread of common urinary pathogens including *Haemophilus influenza* and *Escherichia coli*. However, urinary culture is usually negative. Other rare pathogens reported include Chlamydia trachomatis and *Mycobacterium tuberculosis* [4, 6].

Most cases of the previously reported vasitis patients have histories of surgeries in close proximity to the affected vas deferens. Due to the low prevalence nature of acute vasitis, other risk factors have not been credited to this condition. A retrospective review was conducted by Clavijo et al. in 2010 where the clinical characteristics of eleven patients were thoroughly evaluated [10]. Of the eleven subjects, eight had previous surgeries in the neighboring regions, such as herniorrhaphy, prostatectomy, and perianal fistulectomy. The review also proposed other risk factors such as trauma history, concurrent human immunodeficiency virus/herpes simplex virus infections, and smoking habits [10]. Interestingly, our patient had none of the aforementioned risks factors.

Clinical symptoms include localized pain or palpable mass in the scrotal or inguinal region, some of them with leukocytosis or fever. Acute vasitis could be classification as three groups depending on involved site: scrotal, suprascrotal, and prepubic. It can be easily confused when there is isolated site involvement [11]. The common differential diagnoses include orchitis, epididymitis, testicular torsion, and inguinal hernia. Correct diagnosis is essential because the treatment of vasitis is via antibiotics, and operation is not required [6].

The diagnosis of vasitis could be a challenge by its rarity and unclear image findings. We review past reports that suggest appropriate tools include ultrasound, computed tomography and magnetic resonance imaging (MRI). Ultrasound can be used to exclude orchitis, epididymitis, and testicular torsion by color Doppler. Acute vasitis often reveals heterogeneous, hypoechoic spermatic cord and echogenic fat surrounding the lesions. However, ultrasound is relatively challenging to differentiate from incarcerated inguinal hernia to vasitis [12]. CT and MRI are more recommended to confirm the diagnosis. Spiral CT has high resolution and short scanning time. Acute vasitis typically reveals unilateral edematous spermatic cord without evidence of herniated bowel loop. MRI can provide more soft tissue detail, abnormal signal intensities of inflamed or ischemic structures, with the additional benefits of no radiation exposure [3, 6, 7, 12].

Based on the available literature, the majority of the reported vasitis can be resolved with the use of anti-inflammatories and antibiotics alone [3, 8]. Some authors prescribed oral antibiotics while others started with intravenous antibiotics in combination with oral antibiotics [6]. A reported pediatric vasitis case suggested only partial regression of the inguinal swelling symptoms with 7 days of oral antibiotic use, and the patient was later admitted and treated successfully with intravenous antibiotics [13]. Surgical exploration and drainage may be necessary in more severe cases that are not responsive to antibiotic treatment [10]. En bloc excision of the vas deferens is uncommon and is usually performed for the exceptionally rare cases of Tuberculous vasitis [14]. For our patient, we prescribed empirical antibiotic treatment with 500 mg of Levofloxacin once daily for two weeks. The patient’s symptoms improved drastically upon the scheduled weekly follow-ups.

In summary, we present a rare case mimicking incarcerated inguinal hernia, and although vasitis is very rare, physicians should keep in mind that patients might present with similar symptoms, especially when the patient has risk factors such as previous vasectomy and concurrent leukocyturia. CT or MRI scan can play an important role to prevent unnecessary surgery.

## Abbreviations

CT: Computed tomography
MRI: Magnetic resonance imaging
